# Evaluating Palin Stammering Therapy for School Children (Palin STSC 8–14): protocol for a feasibility randomised controlled trial comparing Palin STSC(8–14) with usual treatment

**DOI:** 10.1186/s40814-022-01158-1

**Published:** 2022-09-16

**Authors:** S. K. Millard, S. Murphy, G. Barton, M. Leathersich, G. Mills, L. Rixon, L. Shepstone, E. Sims, V. Joffe

**Affiliations:** 1 The Michael Palin Centre for Stammering, Whittington Hospital NHS Trust, London, UK; 2 Unversity of Bedfordshire, Luton, UK; 3 Norwich Clinical Trials Unit, University of East Anglia, Norwich, UK; 4 City, University of London, London, UK; 5 University of Essex, Colchester, UK

**Keywords:** Stammering, Therapy, School-aged children, Feasibility, Randomised controlled trial, Usual treatment, Speech and language therapy, Palin STSC(8–14)

## Abstract

**Background:**

Having a stammer can have a significant effect on a child’s social, emotional and educational development. With approximately 66,000 children in the UK having a stammer, there is a need to establish an adequate evidence base to inform clinical practice. We describe a feasibility trial to explore the effectiveness of a new therapy programme for children aged 8–14: Palin Stammering Therapy for School Children (Palin STSC(8–14)). Preliminary data from the Michael Palin Centre, where the programme was developed, indicate that Palin STSC(8–14) is effective in reducing stammering frequency and impact for children, with beneficial effects for parents too. We will investigate the feasibility of the methods required for a definitive randomised controlled trial to investigate the application of this therapy by NHS speech and language therapists (SLTs), compared with ‘treatment as usual’ (TAU), beyond the specialist context in which it was developed.

**Methods:**

This is a two-arm feasibility cluster-randomised controlled trial of Palin STSC(8–14) with TAU control arm, and randomisation at the level of the SLT. Quantitative and qualitative data will be collected to examine the following: the recruitment and retention of therapists and families, the acceptability of the research processes and the therapeutic intervention and the appropriateness of the therapy outcome measures. Assessments will be completed by children and parents at baseline and 6 months later, including measures of stammering severity; the impact of child’s stammering on both children and parents; child temperament, behaviour and peer relations, anxiety; quality of life; and economic outcomes. There will also be a qualitative process evaluation, including interviews with parents, children, SLTs and SLT managers to explore the acceptability of both the research and therapy methods. Treatment fidelity will be examined through analysis of therapy session records and recordings.

**Discussion:**

The findings of this feasibility trial will inform the decision as to whether to progress to a full-scale randomised controlled trial to explore the effectiveness of Palin STSC(8–14) when compared to Treatment as Usual in NHS SLT services. There is a strong need for an evidence-based intervention for school age children who stammer.

**Trial registration:**

ISRCTN. ISRCTN17058884. Registered on 18 December 2019.

**Supplementary Information:**

The online version contains supplementary material available at 10.1186/s40814-022-01158-1.

## Background

Stammering (also known as stuttering) is a speech disorder that is overtly characterised by difficulties in starting, repeating, or prolonging a word or syllable [1]. The speech disruption may also be accompanied by a number of physical behaviours, including facial tension and grimaces, rapid eye blinks and tightness in the upper body [1, 2]. However, it is the less observable features of the disorder which have a greater impact on day-to-day functioning and participation.

Having a stammer can have a significant effect on a child’s confidence, self-esteem and emotional state [3] and is associated with a significantly heightened incidence of anxiety disorders [4]. When persistent, as the stammering continues into school age and adolescence, attitudes towards speech and communication become increasingly negative and the ability to function in everyday speaking situations decreases [5]. School-aged children who stammer are more likely to be rejected, bullied and perceived negatively compared to their normally fluent peers and are more likely to have difficulties establishing friendships with peers than children who do not stammer [6]. Stammering not only has an impact on the child who is stammering, but also affects the parents who express feelings of worry, anxiety, uncertainty, frustration, upset and self-blame [7]. Interactions and relationships between parents and child can also be affected [8].

While stammering is present in all age groups, research indicates that most children who stammer begin to do so before adolescence, generally between 2 and 5 years of age [9]. For some, the experience is transient, but where the stammering persists beyond 18 months post onset or beyond 7 years of age, it becomes increasingly likely that the condition will be chronic [9]. According to the Department for Education (2012) [10], there are 6,614,230 children in state-funded primary and secondary schools in England. Based on a prevalence figure of 1% [9], this means that there are estimated to be over 66,000 school aged children in the UK with a chronic stammer.

In order to improve a person’s quality of life, emotional well-being, social integration, educational attainment and occupational potential, it is critical to provide effective and timely intervention. Providing effective intervention in the school age years has the potential to reduce the emotional and economic consequences of stammering in the adult years. Baxter et al. [11] carried out an NIHR HTA-funded systematic review of non-pharmacological interventions for stammering published from 1990 to 2014. From the 112 papers included, only seven were randomised controlled trials and none focused on school-aged children, the focus of our study. There were studies identified for this age, reporting cross-sectional and uncontrolled trials, but there has been no prospective randomised controlled trial investigating therapy with school-aged children.

There are some quasi-experimental trials and reports of intervention outcomes in the literature. For instance, there is early evidence for the use of an operant-based intervention approach (the Lidcombe Program) [12], syllable-timed speech [13], and prolonged speech, delayed auditory feedback and gradual increase of length and complexity of utterance [14]. These are all speech restructuring or fluency enhancing behavioural approaches to intervention and consequently stammering frequency was the single outcome measure. However, there are a number of challenges to the ecological validity of stammering frequency as the primary outcome measure. Firstly, production of relatively fluent speech using a technique in a test situation does not necessarily reflect generalisation to other contexts or habituation of the skill, and there is little evidence that children maintain or indeed generalise any new speech restructuring method beyond the clinic context. Secondly, increased fluency is not the only, nor indeed the most important, outcome from therapy for children who stammer or their parents [15, 16]. And thirdly, although empirical evidence to support a comprehensive approach is awaited, there is a growing consensus of opinion that therapy should address the cognitive and affective components of the disorder (such as speech related anxiety, frustration, reduced confidence and self-esteem), in order to advance the ability to communicate and participate in daily speaking situations [17].

In addition to the paucity of empirical research, services to children who stammer are affected by inequitable, inefficient and inadequate services for children with speech, language and communication needs [18]. SLTs report reduced knowledge, skills and confidence to work with this client group in particular [19]. The combined effect of all these factors means that services to children who stammer are described as ‘a post-code lottery’, with variability in the content, frequency, context, quality and availability of the intervention available [20].

While there are a small number of specialist centres (e.g. Michael Palin Centre) where children may be able to receive some support when no local support is available, these do not have the capacity to meet the need on a national basis. The current literature shows there is a clear need for evidence-based interventions targeting school-age children which can be delivered by speech and language therapists within *local* services. In order to improve services for children who stammer, further empirical evidence for interventions and increased training for speech and language therapists is required.

This feasibility study will explore potential challenges and aims to establish the viability of a large scale randomised controlled trial to explore the effectiveness and cost-effectiveness of Palin STSC(8–14) as a therapy to meet the needs of children, parents and SLTs. Three preliminary studies have been conducted to establish initial efficacy of Palin STSC(8–14) [21–23] at the Michael Palin Centre. Two describe the outcomes of the therapy delivered during a 2-week intensive group therapy course, with 1-year follow-up data compared to a pre-therapy baseline phase [21, 22]. A third explored the efficacy of Palin STSC(8–14) when delivered through weekly, individual family sessions [23]. All studies reported a reduction in stammering frequency, a reduction in the impact of stammering on the children and reduced parental worry which was maintained for one year post therapy. Parents also reported increased knowledge and confidence to manage the disorder.

These studies indicate the efficacy of this integrated therapy approach when delivered in a specialist context, by therapists who developed the programme and who work with it frequently and intensively with high levels of training and supervision. There has been no study to investigate whether this approach can be taught to, and utilised by, speech and language therapists working outside this context, to improve the lives of children who stammer and their parents. There is no evidence to inform whether this intervention is any more effective or cost-effective than current SLT practices.

This article reports the protocol (V5 16.09.2021) which includes amendments introduced as a result of the COVID-19 pandemic. A number of procedures and methods were altered from the original protocol so that all aspects of the trial could be conducted remotely. These amendments were introduced in order to reduce the requirement for face-to-face contact between researchers, SLTs and families, thereby reducing the risk of infection and protecting the trial as far as possible from the impact of future ‘lockdowns’. Since the onset of the COVID-19 pandemic, the staff at the Michael Palin Centre have demonstrated that Palin STSC(8–14) can be delivered via an on-line platform. While it is too early to evaluate the outcomes of this method of delivery, the feedback from both therapists and clients has been very positive.

### Aims and objectives

The effectiveness of Palin STSC(8–14) in comparison to local services Treatment as Usual will not be assessed in this trial. The aim of this study is to establish the feasibility of a future definitive randomised controlled trial to inform progression to a full-scale trial and indicate any necessary adjustments (changes to procedures, outcome measures, data collection methods, intervention delivery, etc.). This study has the following feasibility objectives:

- *Objective 1*: To determine whether sufficient numbers of SLTs, children and parents can be recruited (via NHS trusts) and retained for a future trial, considering participation, dropout and completion rates.
- *Objective 2:* To examine the range and completeness of the measures used to measure and predict response to treatment. The variability and within cluster (SLT) correlation will be considered with respect to power calculations for the full trial.
- *Objective 3:* To evaluate the acceptability of Palin STSC(8–14) to children, speech and language therapists and parents.
- *Objective 4*: To evaluate the acceptability of the research methods, including randomisation and data collection.
- *Objective 5*: To evaluate treatment fidelity, based on adherence to the therapy programme, by SLTs through quantitative and qualitative assessments. To do this, it will be necessary to develop a treatment fidelity checklist and intervention monitoring documentation.
- *Objective 6:* To explore the feasibility of collecting health economic data.

## Method

### Design

The design is a two-arm cluster-randomised controlled trial with randomisation at the level of the speech and language therapist. Randomisation at the level of the child is not possible due to the number of factors beyond the control of the researchers, which affect whether a specific child is able to access a particular service, location or clinical context (e.g. geographical distance to clinic, service organisation requirements, funding). The sites serve populations that are diverse in terms of socio-economic status, rural vs urban, education levels and ethnicity. Randomising at the level of SLT rather than site is intended to maximise the chance that demographic variables will be spread evenly across the intervention groups and so SLT randomisation will be stratified by site. There will be three participant groups: speech and language therapists, parent-child dyads and SLT managers of services that are participating.

Participating SLTs allocated to the intervention arm will deliver Palin STSC(8–14) and those allocated to the control arm will deliver the usual intervention provided to school-aged children who stammer within that NHS service. SLTs allocated to the Palin STSC(8–14) arm will receive training in the approach immediately. It is possible that their approach to children who stammer will be influenced by this training and so those SLTs allocated to the treatment as usual control arm will have the option to attend training after all study measures have been completed. Potential child participants will be identified by SLTs and will receive intervention based on their assigned SLTs allocation.

**Fig. 1:**
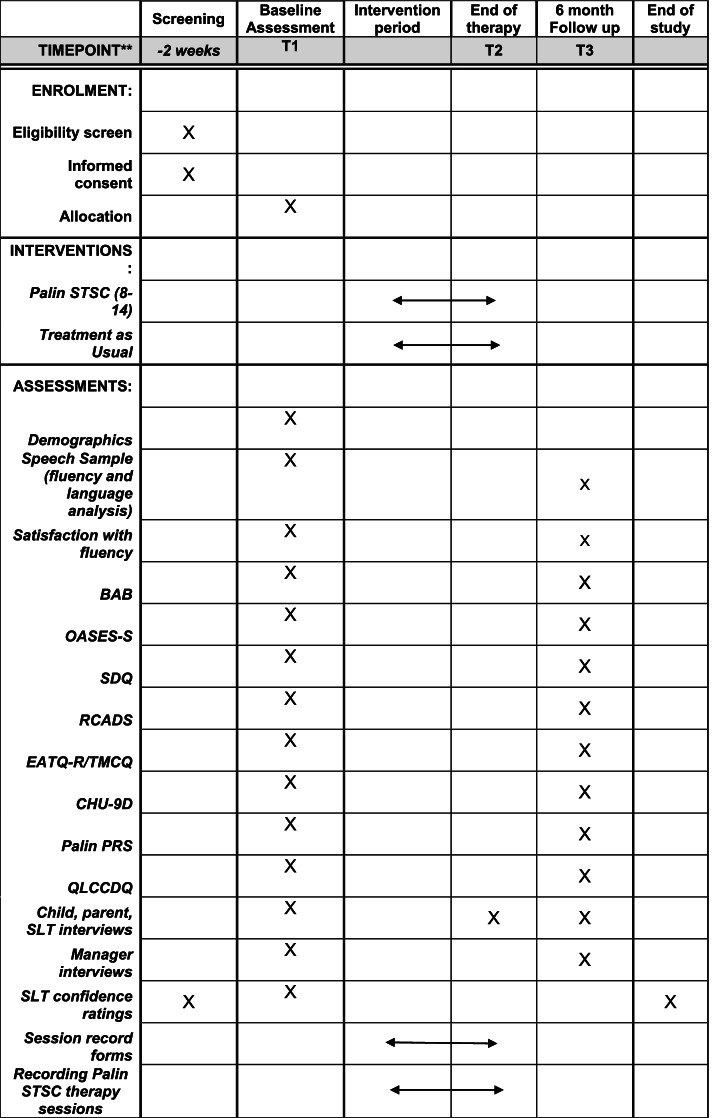
Standard Protocol Items for Interventional Trials (SPIRIT) schedule of enrolment, interventions and assessments for Palin STSC(8–14) feasibility trial

Children and parents will complete questionnaires at baseline and again 6 months later (see Fig. 1). SLTs complete a questionnaire at the start and end of their involvement in the trial. In addition, semi-structured interviews with a purposive sample of SLTs, parents, children and SLT managers will be conducted at the start and end of the trial (all groups) and at the end of therapy (children, parents and SLTs). This trial protocol is in line with Standard Protocol Items: Recommendations for Interventional Trials (SPIRIT) 2013 (see SPIRIT checklist in Additional file 1).

### Setting

This is a multi-site trial. NHS speech and language therapy services in England and Wales which offer, or which have potential to offer, therapy to children who stammer aged 8–14 can take part in the study.

### Intervention

Since the onset of the pandemic in March 2019, there has been an increase in access to services through telehealth. To reflect this change, either intervention may be accessed during face-to-face appointments or via a video platform.

#### Treatment as usual

There is no defined or accepted standard practice for this client group and little evidence of what constitutes Treatment as Usual and so the intervention that children receive will vary across services. It may include generalist therapy, group therapy, reviews of progress, referral to a specialist service or no treatment at all. It is anticipated that the goals and content of therapy will also differ and may include general advice, fluency strategies, stammering modification, confidence speaking and/or increased environmental support.

#### Palin stammering therapy for school children (8–14)

The primary aim of the intervention is for the child to become a more confident and effective communicator. In order to achieve this, the following goals are targeted:

- Maximising the child’s fluency and/or reducing struggle speaking
- Enhancing communication skills in the family
- Increasing confidence and participation in speaking situations
- Reducing child’s and parents’ worry about stammering
- Developing parents’ skills and confidence to support the child

Sessions start with a Solution Focused Brief Therapy session to explore goals and expectations from therapy [24]. Therapy focuses on the development of skills across three main areas: (1) family communication skills, (2) strategies to address the cognitive/emotional needs and (3) direct fluency work. The approach assumes that the child is only able to make changes in these areas with the support and input of at least one parent/carer and so they are involved in the therapy throughout. Palin STSC(8–14) comprises 10 sessions, as this ‘dosage’ level was shown to be efficacious in our preliminary study [23].

### Participants

There are three groups of participants: SLTs who deliver the intervention, parent and child dyads and SLT managers. No formal sample size calculation was carried out. For the purposes of this study we have used the available literature and discussions with local SLT services to provide some estimations of recruitment and response rates. We seek to recruit a total of 30 SLTs to the trial (i.e. 15 per intervention arm), with each therapist providing on average 2 children (and no more than 4). We aim to recruit in total 60 children who stammer between the ages of 8 and 14. With the expectation of attrition of around 20%, this would result in a total of around 50 completers. We will recruit 4-6 SLT managers to be interviewed.

#### Recruitment

*SLTs:* SLTs will be recruited from NHS speech and language therapy services who receive referrals for children who stammer and are aged between 8 and 14 years old. The services can be located anywhere within England or Wales in line with the trial’s ethical approvals from the Health Research Authority.

SLTs will be made aware of the study through personal contact, social media posts, notifications through Clinical Research Networks, SLT special interest groups and through the Royal College of Speech and Language Therapists. No previous expertise or experience in stammering is required. SLTs and managers will be sent SLT participant information sheets and a trial summary in response to any expression of interest.

**Fig. 2:**
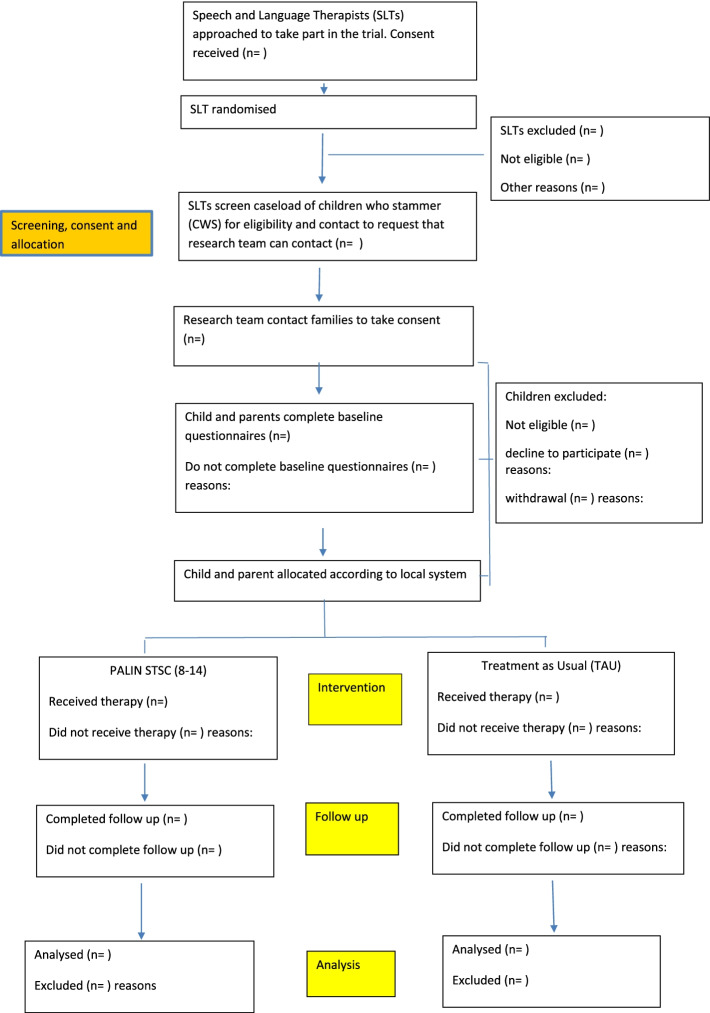
Palin STSC flowchart

*Child and Parent participants: *The participating SLTs will be responsible for identifying eligible children who are on the caseload or who are referred to their service for stammering intervention. Figure 2 summarises the process of recruitment and participation for children and parents. Once identified by a SLT, the parent will be approached by the SLT to be told about the service’s involvement in the trial and to request their consent to be contacted by the study research team. The study team will then contact the parent to screen for eligibility and initiate the informed consent process. The Participant information sheets, for both children and parents, as well as copies of the consent and assent forms will be sent for consideration and 2 weeks will be given for the family to consider participating.

*SLT Managers:* A purposive sample of 4-6 managers of services that are participating in the trial will be invited to take part in semi-structured interviews.

#### Consenting

*SLTs:* SLTs will be offered time with the research team to discuss the trial and to ask questions. If the SLT wishes to proceed and the service manager supports this, then the research team will liaise with the local R&D team to open the trial at that site. SLT consent will be taken remotely via the trial database.

*Children and parents:* At the end of a 2-week consideration period, the family will be contacted by the research team to provide an opportunity to ask questions. The parent consent form and child assent form will be completed remotely via the database.

#### Inclusion criteria

The inclusion criteria for each participant group is as follows:

- *SLTs*:
  - Not currently delivering Palin STSC(8–14) and not previously trained in the approach
  - Have the potential to identify and provide intervention (either Palin STSC(8–14) or Treatment as Usual) to at least 2 children within the duration of the study.
- *Children:*
  - Aged between 8 years 0 months and 14 years 11 months at the start of the study
  - Diagnosed/recognised as stammering by speech and language therapist, parent and self.
  - Want to receive stammering therapy
  - Have at least one parent who is able to attend therapy and participate in the study
- *Parents:*
  - Have a child who is participating in the study
- *SLT managers:*
  - Have management responsibility for the SLTs involved in the study
  - Had a role in the decision about whether the service would take part in the trial

#### Exclusion criteria

- *SLTs:*
  - Currently working with families on a weekly basis using principles of Palin STSC(8–14)
  - Unable to provide weekly family therapy sessions if allocated to Palin STSC(8–14) due to service or resource limitations.
- *Children:*
  - Have received therapy in the previous 6 months or are currently receiving speech and language therapy
  - Have attended the Michael Palin Centre for assessment or therapy
  - Do not have a parent who consents to take part in the study
  - Are involved in any other research study which involves an intervention or multiple assessments
  - Are accessing formal counselling through psychological services and the addition of Palin STSC is contra-indicated
- *Parents:*
  - Their child has been excluded for any reason
  - There are any contra-indications for therapy. This might include any significant issues in the family which would be taken into account for the timing of any therapy, e.g. bereavement or birth of a baby.

### Randomisation and allocation process

*SLTs:* The randomisation list has been generated by Norwich Clinical Trials Unit data management team using an online sequence generation service, which is reviewed and approved by the study statistician. Speech and language therapists (SLTs) are randomly allocated into one of the two treatment arms: Palin STSC(8–14) or TAU, using blocked randomisation with a block length of 2, stratified by service.

Following consent and baseline data collection, a member of the trial team responsible for trial enrolment randomises the SLT via a web-based randomisation system integrated into the study database. An immediate allocation is provided by the system to the person entering the data. Automated email alerts are sent to the Site PI and the Research Assistant informing them of the allocation.

*Child and parent dyads*: Once baseline assessments are completed, families will be allocated to a SLT in accordance with local SLT service procedures. For some, the SLT is predetermined by clinic or school attended; for others, families are allocated on the basis of SLT availability (i.e. when a therapist has a free assessment appointment, the next family on the list are allocated to that appointment and therapist). Processes were monitored by the research team to avoid the potential of bias in recruitment and allocation processes.

### Procedure

#### SLT training

*TAU:* SLTs in the TAU arm will receive training on the research process and procedures.

*Palin STSC(8*–*14):* SLTs in the Palin STSC(8–14) arm will also receive research protocol training, along with training in the Palin STSC(8–14) assessment and therapy methods. This has been developed into a package of 13 video-recorded sessions which take approximately 20 h to complete (comparable to the original 3-day face to face training programme). In addition, SLTs will be able to attend up to three remote question and answer sessions with an SLT who is specialist in the programme.

#### Fidelity assessment

SLTs from both arms will complete an online ‘session summary checklist’ following each therapy session to detail the logistical information (location, attendees, session number, length of time, etc.) and therapeutic elements/topics covered. Those from the Palin STSC(8–14) arm will be used to check the topics covered in each session for fidelity purposes. In addition, SLTs delivering Palin STSC(8–14) will video record each therapy session. A random sample of two sessions from each child will be evaluated by an SLT who is a specialist in the programme, using a treatment fidelity checklist to be developed as part of the trial.

### Measures

Basic demographic data on age, gender and ethnicity will be collected alongside stammering history information (time since onset, family history, previous therapy history). Selected quantitative measures have been chosen for their psychometric properties and clinical utility. Measures will be used to evaluate the feasibility outcomes: to ascertain recruitment and retention, treatment fidelity, treatment outcome, acceptability of the research and therapy methods. The study also includes measures for variables that are predicted to influence stammering impact and therapy outcome, since this will form part of the full-scale trial. Some of the assessments completed are directly relevant for the implementation of therapy and the results will be provided to the therapists for their clinical decision-making needs (those provided to therapists in the Palin STSC(8–14) arm are marked *). Each therapist providing TAU will identify in advance any assessments that are part of usual practice and will receive the results of those. The online and postal questionnaires will take each parent and child approximately 1.5 h to complete.

#### Recruitment and retention rates

Recruitment and attrition rates will be established for SLTs, children and parents. The numbers of children approached, numbers consenting and attrition rates will be recorded. Participants are free to withdraw at any time without giving reasons and without prejudicing any further treatment that would be usually available. Participants will be asked if they are willing to say why they are withdrawing so that this information may be used in the context of whether the therapy and trial methods are acceptable. Permission will be sought to use data to the point of withdrawal but this may be withdrawn up until the time of analysis.

#### Child-completed measures

##### Speech sample*

A video recording of the child reading and talking during conversation, and analysed by the research assistant using the Stammering Severity Instrument Fourth Edition [25] (SSI-4), provides a standardised clinical measure of stammering severity. The conversation section will be analysed using the CHILDES [26] system to examine the child’s use of language and turn taking skills. This will be conducted at both baseline and follow-up (4 min).

##### Satisfaction with fluency [27]

Adapted from the Satisfaction with Communication in Everyday Speaking Situations Scale [27] and completed on the on-line database. Children rate “how happy are you with your speech at the moment?” on a 9-point scale at both baseline and follow-up (1 min).

##### Behavior Assessment Battery [28]

The Behavior Assessment Battery [28] includes Behavior Checklist, Speech Situation Checklists and Communication Attitude Test completed on paper. The Behavior Checklist requires a yes/no response to a series of potential features of stammering. The child records whether or not each is typical of their own speech. Speech Situation Checklist consists of two parts. In the first, the child rates the severity of the stammering in different speaking situations (SSC-SD) on a series of likert scales, and the second, the child rates the degree of fear about speaking in different situations (SSC-ER). Communication Attitude Test-Revised (CAT-R)* evaluates the speech-related belief system of children who stammer using 35 items with Yes/No response. These assessments will be completed at both baseline and follow-up (30 min).

##### The Overall Assessment of the Speaker’s Experience of Stammering (School age/Teenage versions) (OASES-S/T) [29]*

The OASES-S [29] evaluates the impact of stammering on an individual’s life across four sections: general information, reactions to stammering, communication in daily situations and quality of life. For participants over the age of 12, the teenage version will be used. The OASES-S or OASES-T will be completed at both baseline and follow-up (15 min).

##### Strengths and Difficulties Questionnaire (SDQ) [30]

This is a screening tool for behavioural, emotional and social development with 5 subscales: emotional symptoms, conduct problems, hyperactivity/ inattention, peer relationship problems and pro-social behaviour. Children aged 11–14 years will complete an adolescent version at both baseline and follow-up (10 min).

##### Revised Children’s Anxiety and Depression Scale (RCADS) [31]

This is a 47-item, youth self-report questionnaire with subscales including separation anxiety disorder (SAD), social phobia (SP), generalised anxiety disorder (GAD), panic disorder (PD), obsessive compulsive disorder (OCD) and major depressive disorder (MDD). There are parent and child versions for each to complete at both baseline and follow-up (10 min).

##### Early Adolescent Temperament Questionnaire-Revised (EATQ-R) [32]

Designed to assess temperament in 9–15 year olds to gain insight into positive and negative reactivity and regulation. There are two questionnaires, one for parents and one for young people. In the definitive trial, temperament will be considered as a potential predictor variable for outcome. The EATQ-R [32] will be completed at baseline only.

##### Child Health Utility 9D (CHU-9D) [34]

This is a series of 9 questions for the child to rate how they feel and their ability to participate in daily activities. The scores from this assessment can be converted to quality-adjusted life years and will be used in the health economic evaluation and it will be completed at both baseline and follow up (10 min).

#### Parent completed measures

##### Palin Parent Rating Scales (Palin PRS) [35]*

This standardised assessment consists of 19 rating scales and is completed by parents to measure their perspective across three factors: the impact of the stammer on the child, the severity of the stammer and the impact on the parents and parents’ knowledge about stammering and their confidence to support their child. It will be completed at both baseline and follow-up (5–10 min).

##### Strengths and Difficulties Questionnaire (SDQ) [30]

Parent version of the SDQ described above will be completed at both baseline and follow-up (5–10 min).

##### Revised Children’s Anxiety and Depression Scale (RCADS) [31]

Parent version of the RCADS described above will be completed at both baseline and follow-up (10 min).

##### Early Adolescent Temperament Questionnaire-Revised (EATQ-R) [32]

Parent version of the EATQ-R described above. Parents of children aged 8 complete the Temperament in Middle Childhood Questionnaire (TMCQ) [33]. This will be completed at baseline only (40 min).

##### Quality of Life in a Child’s Chronic Disease Questionnaire (QLCCDQ) [36]

This is a 15-item Likert scale concerning the impact of a child’s impairment on a parent’s life. Parents will rate the degree to which their daily lives have been affected by the child’s condition in the previous 2 weeks, at both baseline and follow-up (10 min).

##### Service Use Schedule

This questionnaire has been developed for the purposes of this study and explores the use of different services for the health economic evaluation. It will be completed at both baseline and follow-up (10 min).

#### Completed by speech and language therapists

##### Confidence and knowledge about working with children who stammer

A questionnaire has been developed for the purposes of this study to explore therapists’ knowledge and practices for working with school age children who stammer in the NHS. The SLTs confidence in working with this client group will also be explored. It is anticipated that these factors will improve with the trial for therapists in both treatment arms and so this will be completed at baseline and at the end of the trial. Those in the Palin STSC(8–14) treatment arm will also complete this at the end of their training in the programme (15 min).

##### Session record form

SLTs will record the content of each therapy session using a checklist, along with attendance and the homework completed by the family. This will inform the degree to which SLTs are completing the programme and the engagement of the families, providing data for the treatment fidelity evaluation and acceptability of the therapy methods (10 min per therapy session).

### Data collection

Each parent and child dyad will complete the questionnaires at baseline and again approximately 6 months later. Study data will be collected and managed using REDCap electronic data capture tools hosted at the University of East Anglia [37]. REDCap (Research Electronic Data Capture) is a secure, web-based application designed to support data capture for research studies, providing (1) an intuitive interface for validated data entry, (2) audit trails for tracking data manipulation and export procedures, (3) automated export procedures for seamless data downloads to common statistical packages and (4) procedures for importing data from external sources.

Prior to COVID, all measures were completed online into a REDCAP database platform developed and managed by NCTU with the exception of the Speech Situation Checklists, Communication Attitude Test [28], Strengths and Difficulties Questionnaire [30] and Stuttering Severity Instrument-4 [25] which were completed in person with the Research Assistant. Following COVID, the Speech Situation Checklists, Communication Attitude Test [28] and Strengths and Difficulties Questionnaire [30] were changed to postal completion and the Stuttering Severity Instrument-4 [25] was collected via video link with the Research Assistant.

All SLT measures were completed online.

### Process evaluation

As recommended by the Medical Research Council guidance for RCTs [38], we will include a qualitative process evaluation of the trial and the intervention. We hope to identify the barriers and facilitators to taking part in the trial, as well as modifications that can be made to improve the experience of taking part in this research.

A purposive sample of 4 SLTs, 4 children and 4 parents from each treatment arm will be recruited to take part in semi-structured qualitative interviews at the start and end of therapy, and at the end of the trial. We will seek to recruit children of different ages, from different services, with varying levels of stammering impact, and parents with differing levels of knowledge and confidence. We will select therapists from different speech and language therapy services and from both Palin STSC(8–14) and Treatment as Usual groups. These participants will complete semi-structured interviews at the three time points (total: 72 interviews).

We noticed that when we first spoke to SLTs and SLT teams that they were very enthusiastic and would suggest that multiple SLTs would be available to take part. However, it was often the case that by the time the R&D approvals were in place and recruitment of SLTs started, the numbers reduced considerably compared to the numbers originally proposed. Further, during conversations with SLTs during their baseline interviews and in their training sessions, it became apparent that the support and attitudes of different managers varied but crucial for successful participation. We therefore amended the protocol to include interviews with managers to identify the drivers that influenced decisions about whether a service or individual therapist could take part in the trial and the amount of time they would be given to do so. Understanding the facilitators and obstacles from managers’ perspectives will inform how managers are approached and the information that they receive in the full-scale trial.

We will interview a small sample of 4–6 service managers of participating sites at two time points: the opening of their service as a research site and at the end of the trial. We will aim to understand more about why the service managers agreed for their service and staff members to take part in the trial, their expectations of service participation and their experience of having their service involved. All interviews will be conducted over the phone and audio recorded.

Once the Palin STSC(8–14) training package is completed, SLTs will provide feedback about the quality and content of the training, with recommendations for any changes. This is particularly important given the changes made to the method of training that were made as a result of the pandemic (15 min).

### Analysis plan

The primary statistical analysis will be directed towards the aims of the feasibility study outlined under the aims and objectives section. Particularly, the values under objective 1, including recruitment and attrition rates, will be calculated with an appropriate 95% confidence interval to inform the design of a future study. Similarly, we will aim to quantify and summarise treatment ‘doses’ actually delivered and adherence.

Although not designed as an efficacy trial, we will make an initial estimate of treatment effect on the above-named efficacy variables using linear models with appropriate link and error terms. These will adjust for known baseline prognostic variables (decided prior to analysis) and will be presented with 95% confidence intervals. Data from all participants as randomised in the trial will be included in the ‘intention to treat’ analyses. To avoid attrition bias, outcome data from all participants will be included regardless of protocol adherence.

For the process evaluation, all interviews will be transcribed and analysed using constant comparisons from grounded theory methodology to explore themes regarding process variables, fidelity and acceptability for both the therapy and the trial methods.

In terms of health economics, estimation of cost-effectiveness, within a health-technology assessment, is an iterative process [39]. Here we aim to monitor levels of resource-use and quality of life (QoL), to inform the decision as to how costs and benefits can be measured as part of a future, more definitive study. We will therefore estimate the completion rates for both the aforemtioned CHU-9D (responses to the CHU-9D can be converted into QALY (quality-adjusted life year) scores) [40] and service use schedule, and seek to identify big cost drivers.

## Discussion

The aim of this study is to examine the feasibility of a definitive randomised controlled trial comparing Palin Stammering Therapy for School Children (8–14) with treatment as usual. The programme was developed to meet the needs of school aged children who stammer and their parents and to provide speech and language therapists with an evidence based programme that they can implement with confidence following training. While the principles of the intervention appear to be theoretically sound and the clinical outcomes obtained at the centre where it was developed are promising, there are many questions about the transferability and effectiveness of the programme into non-specialist NHS contexts.

The first aim of the feasibility study is to establish whether sufficient numbers of SLTs, children and parents can be recruited to a full trial. We need to establish attrition rates at each stage in order to establish the sample size required for statistical power in the full trial. We wish to identify the factors that affect recruitment and attrition to adjust the procedures accordingly for the definitive trial, where possible.

The second issue relates to the quantitative battery of measures included. The measures have been selected for their potential to provide estimates of the effectiveness and the cost-effectiveness of the intervention and the factors that predict outcome. The overt characteristics of stammering would be expected to reduce with this therapy, either in terms of frequency or severity (amount of struggle and duration, along with frequency) and is measured through the speech sample analysis. However, there are important limitations to stuttering frequency and severity measures as they do not necessarily reflect the degree to which stammering is occurring. People who stammer frequently avoid and change words or use strategies to mask the stammering. Also, what might be considered by a listener to be ‘mild’ may be experienced differently by the speaker. Furthermore, as children reduce avoidance behaviours, overt stammering would be expected to increase and would be considered a positive outcome. Therefore, it is important to seek the speaker’s view of their stammering behaviour alongside objective observational methods. In addition, stammering can affect a person’s ability to communicate and participate in daily situations and can have an impact on emotional well-being. Stammering also has an impact on parents. Reducing the impact of stammering is an important outcome for therapy. However, the impact that stammering has on either a child or parent is not necessarily related to the frequency or severity of the stammering and so this has to be measured separately and so there are a number of measures included in this trial for that purpose (OASES-S [29], BAB [28], Palin PRS [35]). Finally, the impact of stammering and the outcome of therapy are influenced by a number of variables, which may or may not include stammering severity and so other, more distal variables likely to be associated with therapy outcome (e.g. temperament [41]) will also be completed. In this feasibility trial, we aim to find out if this battery of assessments is sensitive and extensive enough for the outcome evaluation, but we are also aware of the potential burden of such an extensive battery. It will be important to establish whether the assessment battery is too large, creating a burden that contributes to recruitment and retention rates.

The remaining issues relate to the acceptability of the therapy approach and the trial methods, including fidelity and adherence to the programme. To date this programme has only been implemented at the Michael Palin Centre where it was developed, by therapists who are highly specialist in the field and who have been involved in the development of the programme. It is not known whether therapists with less background knowledge and experience in stammering can be taught these methods and gain a strong grasp of the underlying principles of the intervention through a relatively short training programme. It is not known what therapy or how much therapy is currently available to this client group and whether therapists will be able to integrate this programme into their working practices. It is also possible that outcomes may be affected by any differences between local populations and the population who attend the Michael Palin Centre, such as levels of complexity or motivation. Any of these unknown variables will influence the implementation of the definitive trial and therapy outcomes in the NHS in the long term.

Since opening in June 2019, there have been some substantial amendments to the trial methods. The trial was suspended in March 2020 as a result of the COVID-19 pandemic. It re-opened in January 2021 following a consultative scoping exercise with recruited sites. Speech and language services were severely affected by the pandemic, many therapists were redeployed, some services were closed entirely and others offered only emergency support. Those sites that had opened prior to the pandemic were largely located in and around London and were particularly badly affected. Despite intentions to reopen, the ongoing and unpredictable nature of the pandemic meant that recruitment of sites and therapists had to start again, almost from the beginning, when the trial reopened. Prior to the trial re-opening, all procedures were amended so that they could take place remotely. This was to eliminate any additional face-to-face contact incurred through the trial and to try to ensure that the trial could proceed if further lockdowns occur. The changes mean that consent is completed via the online database, research assistant-led assessments are conducted by a video call or sent by post, SLT training has been converted into a series of video recordings that can be completed over time and there is an option to deliver therapy via an online video platform. These changes resulted in a number of unexpected advantages with procedures which are considerably more resource efficient for SLTs, families and the research team, increasing accessibility to both therapy and the research trial, and enabling recruitment to extend beyond the South-East of England.

If there is evidence from this feasibility trial that SLTs, parents and children are willing and able to participate; if the barriers to recruitment and retention can be identified and addressed; the outcome measures are deemed appropriate and sensitive to change; and, the therapy and research methods are acceptable to participants, then this protocol will be adapted and developed into a full scale definitive trial. Ultimately, should the study progress to a definitive trial and demonstrate effectiveness, the benefits to children who stammer and their parents could be substantial. Having an evidence-based intervention that can be implemented in local services has high potential to influence SLT training, inform service delivery models, increase SLT confidence in working with this client group, improve outcomes and reduce the long-term impact that stammering can have on children and their parents.

### Trial status

Ethical approval was obtained on 17 June 2019 from London-Bloomsbury REC. Following suspension of the trial through the pandemic, a funded extension was received. Recruitment of SLTs was closed early as the target numbers were reached and recruitment of children and parents will be completed by December 2021 to allow for follow-up data collection by 6 June 2022. Intervention is currently underway. Data analysis and interpretation of the results will take place before the end of the trial on 31 August 2022.

### Adverse events

As this is a low-risk trial, a clinical review of a line listing of all SAEs, AEs, ARs and trends in events and reactions will take place on a monthly basis by the Research Team (i.e. those involved in the day to day running of the trial). Any adverse events or reactions which are considered serious by the PI or SLT involved will be reported to the CI within 24 h.

A cumulative review of all safety information will be conducted on a 6 monthly basis by the Research Management Group and the Trial Steering Committee.

### Monitoring

There is an Advisory Group made up of the research management group and members who represent parents of children who stammer; charity groups who advocate for people who stammer (Action for Stammering Children; Stamma); generalist and specialist SLTs; and young people who stammer who have sought SLT through the NHS in the past. There is a Trial Steering Committee (TSC) made up of independent experts who oversee the implementation and progress of the trial. The decision to stop the trial prematurely will be made by the Trial Steering Committee and the Sponsor if fewer than 25% of participants have been recruited 1 year into the trial, or if there is substantial evidence that participants in the Palin STSC(8–14) group are withdrawing as a consequence of the therapy itself.

Both the TSC and the Advisory group meet on a 6 monthly basis or more frequently as required. Site procedures will be monitored on a monthly basis by the CI, the RA and the local PI. Procedures will be monitored 3 monthly by the research management group and six monthly by the TSC. Trial processes undertaken by the RA will be reviewed once a month by the CI and RMG during the data collection phases of the study, and six monthly by the TSC.

### Dissemination

We aim to publish the methodological lessons and quantitative and qualitative findings in peer-reviewed scientific journals. Preliminary results will be presented at the International Fluency Conference May 2022. Participants (SLTs and parents) will be notified of the outcome of the trial, via a specifically designed summary report. Per standard NIHR contractual terms, the trial’s funder will have authority over publication. Whittington Health, as Sponsor, will not have authority over publication. The participating investigators will have rights to publish the trial data if agreed by the CI and co-collaborators. Professional writers will not be used.

## Supplementary Information

**Additional file 1.** SPIRIT checklist.

## Data Availability

No data were generated or analysed for this protocol. Direct access to documentation and materials will be granted to authorised representatives from the Sponsor, host institution and the regulatory authorities to permit trial-related monitoring, audits and inspections—in line with participant consent.
